# The viable circulating tumor cells with cancer stem cells feature, where is the way out?

**DOI:** 10.1186/s13046-018-0685-7

**Published:** 2018-02-26

**Authors:** Y. T. Luo, J. Cheng, X. Feng, S. J. He, Y. W. Wang, Q. Huang

**Affiliations:** Molecular Diagnostic Laboratory of Cancer Center, Shanghai General Hospital, School of Medicine, Shanghai Jiao Tong University, Shanghai, 201620 China

**Keywords:** Circulating tumor cells, Culture, Expansion, Function

## Abstract

With cancer stem cells (CSCs) became the research hotspot, emerging studies attempt to reveal the functions of these special subsets in tumorigenesis. Although various approaches have been used in CSCs researches, only a few could really reflect or simulate the microenvironment in vivo. At present, CSCs theories are still difficult to apply for clinical remedy because CSCs subpopulations are always hard to identify and trace. Thus an ideal approach for clinicians and researchers is urgently needed. Circulating tumor cells (CTCs), as the method of noninvasive-liquid biopsy, could be detected in the peripheral blood (PB) from many tumors and even could be treated as procurators for CSCs deeper researches from patient-derived sample. However, CTCs, as a diagnostic marker, also raise much controversy over theirs clinical value. Mechanisms causing CTCs to shed from the tumor have not been fully characterized, thus it is unclear whether CTCs represent the entire makeup of cancer cells in the tumor or only a subset. The heterogeneity of CTCs also caused different clinical outcomes. To overcome these unsolved problems, recently, CTC researches are not just depend on enumerations, whereas those CTC subsets that could expand in vitro may play a pivotal role in the metastatic cascade. Here, we retrospect the CTC developmental history and discourse upon the enrichment of viable CTCs in functional assays, probe the further avenue at the crossroad.

## Background

For decades, tumor formation and development has been regarded as a mysterious issue, compelling scientists to seek the mechanism of origin. Much evidence hinted that some small subpopulations of tumorigenic cells were the causation of tumor recurrence and metastasis, but it may be difficult to draw definitive conception because of the lack of rigorous model and effective methods to identify these special subpopulations. Since 1960, when the Philadelphia (Ph) chromosome and its unique association with chronic myeloid leukaemia (CML) were discovered [1], evidence has been found that the appearance of clonal chromosomal aberrations caused abnormal cell proliferation in bone marrow. These pathological cells could be the culprit of tumorigenesis. Further research also found that these cells (Ph+) were always detected in circulation [2]. From then, cells with special markers had been noticed by researchers. The concept of cancer stem cell (CSCs) began to appear in the mid-1990s by isolating rare cells in the blood of patient with leukemia, these cells were capable to grow into a new leukemia when injected into mice [3]. The early discoveries contributed CSCs to become the hotspot and thus diverse CSC models were emerged subsequently. Many studies provided proof for the CSC hypotheses and managed to address and deduct the process of tumor initiation and development. Unfortunately, these relative hypotheses had not got the final conclusion [4–7], none could perfectly illustrate the details of every step in tumorigenesis and its relapse. It is still unknown about which CSCs paradigm is really suitable for modern clinical therapy. And now to solve these unsettled arguments, more researchers expect to focus on a single-cell level, which could have more convincing to reveal mechanisms of CSC. Therefore, the development of single-cell diagnostic methods is flourishing these years. Circulating tumor cells (CTCs) in the peripheral blood (PB) from different types of tumors are increasingly detected by various methods. However, the mechanisms causing CTCs to shed from the tumor have not been fully characterized, thus it is unclear whether CTCs represent the entire makeup of cancer cells in the tumor or only a subpopulation [8]. Nevertheless those CTC subsets, with CSCs feature, could expand in vitro may play a pivotal role in the metastatic cascade.

## The viable CTCs with CSCs feature for functional analysis

**Fig. 1:**
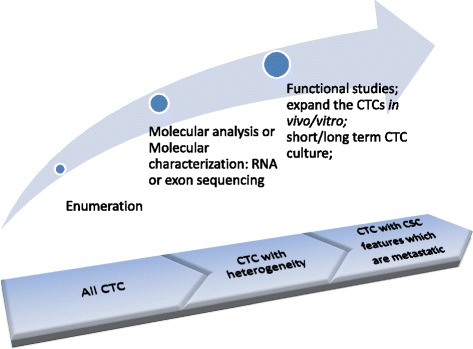
CTC researches undergone the three stages CTC enumerations include various subsets such as dormant cells, apoptotic cells, and even normal hemopoietic stem cells, only depend on enumeration is not suitable for clinical evaluation. Whereas, further the studies in molecular characterization by RNA or exon sequencing could explain CTC heterogeneity, expanded CTCs could be the special subsets not only for deeper molecular-level researches but for functional analyses and guide the clinical therapy

**Fig. 2:**
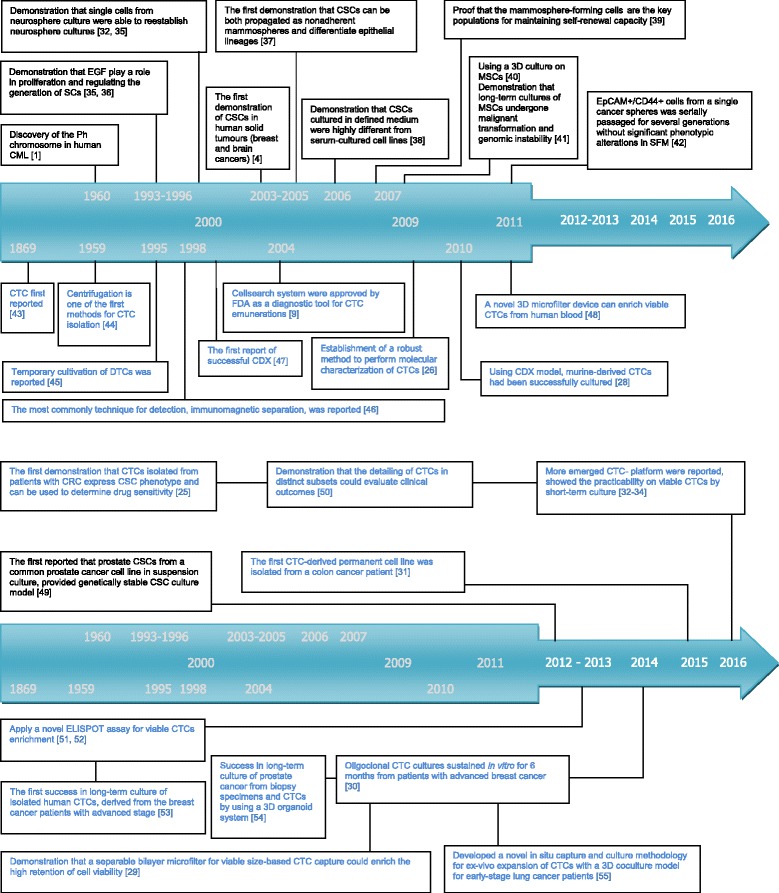
The most influential events contributing to the causal relationship between CSCs and CTCs The fonts in black indicate events related to CSCs [1, 4, 32, 56, 57, 62–68]. The fonts in blue indicate events related to CTCs [9, 26, 28–34, 48, 54, 69–78]. And recent years, many evidence showed the inextricable connection between CSCs and CTCs, the expanded CTCs subsets are always used as a tool to reflect intrinsic characteristic of CSCs [25, 77, 78]. The abbreviations in Fig. 2: stem cells (SCs); mesenchymal stem cells (MSCs); serum-free medium (SFM); disseminated tumor cells (DTC); CTC-derived xenografts (CDX) [35–39, 43–48, 51, 52, 54]

Since the CellSearch system was designed to detect detached tumor cells in PB, CTCs enumeration was thought to be an important method in the clinic relevance [9]. However, there were some limitations for CTC applications. The one was that the released CTCs number in different tumor types were quite disparity [10]. For example, inflammatory breast cancer (IBC) is characterized by high vascularity and increased microvessel density which may increase the chance for the CTCs release [11]. The higher incidence of CTC has also existed in SCLC patients with COPD, the inflammatory conditions and accumulation of airway macrophages which construct particular niches and enhance the invasive ability of CTCs to degrade the extracellular matrix (ECM) in early stage [10, 12] than other cancer types.. Apparently, a threshold of 3–5 CTCs/7.5 ml blood has been defined by the CellSearch system for prognostic stratification [10], which seems not compatible with all cancer patients. Other limitations were that enriched CTCs could not accurately cover the whole population and not all CTCs detected are clinically relevant [13]. Many isolated methods for CTCs relied on either defined surface marker or differences in the size of individual cell populations [13–16]. But CTCs are not a homogeneous group that can be captured by a set of identical markers or the same physicochemical properties. A few CTCs could remain the vitality in a very hostile environment during circulation [13, 14, 17, 18] by fusing with bone marrow-derived cells or altering the phenotype that could protect and hide them from the immune system attack. The methods based on the CD45 marker- even be considered that only express in mature mononuclear blood cells, was found that could even be appeared in CTCs by adhering to platelets or recruiting macrophages [10, 19]. And assumed epithelial markers, such as EpCAM, could also miss the CTCs subsets with low or absent expression [20] and inevitably cause decreased detection of CTCs that had undergone epithelial-mesenchymal transition (EMT), an important alteration involved in metastasis [13]. Contrary to transient disseminated tumor cells (DTC), these altered CTCs may be the key subsets which could manifest CSC features and significantly correlated to treatment response [15, 21–23]. In order to overcome these limitations, some specific markers that have a high specificity were used to define certain tumor types, such as mammaglobin for breast cancer and prostate-specific antigen (PSA) for prostate cancer. Nevertheless, these markers could be also downregulated during dedifferentiation of tumor cells [24] or absent in some particular CTCs due to the heterogeneity and plasticity [15]. These dynamic changes hindered CTC as a biomarker for clinical applications. For extending the understanding of relevant CTCs involved in metastasis, fortunately, the molecular technologies had integrated into CTCs identification by single-cell analyses such as RNA or exon sequencing [25, 26], which could be used to perform quantitative gene expression profiling for special CTCs and potentially guide patient management [26]. However, although studies of molecular characterization did identify different CTC subpopulations within a single blood sample, they had not addressed the biology of CTCs due to the scarcity of CTCs in the PB [25, 27]. To solve this issue, techniques on CTCs expansion both in vitro and in vivo had appeared (Fig. 1). The “viable CTCs”, which were enriched and isolated by label-free methods based on biophysical rather than biochemical properties, became the important role in experimental functional assays. One study reported that isolated human CTCs from murine blood showed an enhanced aggressive phenotype under hypoxic environment in vitro and in vivo [28]. The produced viable CTCs from xenografts in mouse manifested more biologic activity for functional researches. Other study also defined that qualified enrichment of viable CTCs must include some important parameters, such as capture efficiency, enrichment rate and even cell viability [29]. Recently, several groups have achieved a huge harvest in the expansion of CTCs from cancer patients. Two papers reported patient-derived CTCs culture for 6 months [30] and 1 year [31] respectively. Sufficient viable CTCs as a procurator for CSCs functional analyses could provide more biological information. But the next challenging obstacles had also existed. Many researchers concerned issues that were the efficient establishment of human-CTC cultures and the value for clinical applications. Recent years, the study reported that CTCs with CSCs phenotype derived from colorectal cancer patients could be designed to test drug sensitivity and integrate a personalized approach to clinical utility [25]. And then, much more CTC-platform provided the practicability on separation of viable CTCs by subsequent short-term growth in culture [27, 32–34] for functional test of CTC lines. Success in culturing human CTCs would overcome the difficulty of characterizing these rare cells and could extend new potential therapeutic strategy (Fig. 2).

## Functional analysis of CTCs revealed modern individual treatment

Traditional CSC models suggested that there were intratumor heterogeneity in the primary site due to some special tumor cells get gene mutations which were able to become CSCs subpopulations and resulted in the tumor recurrence, metastasis or chemical drugs resistant. Current opinion even believed that these CSCs subpopulations were not immutable [16, 35–38]. Theoretically, under different environmental stress, CSCs and non-CSCs subpopulations were in a dynamic conversion [16]. Owing to the challenge of identify CSCs subpopulations, CTCs as a “monitoring method” were often used to study on the heterogeneity of CSCs in patient-derived samples in real-time. Some researchers had found CTCs and parental cells or primary tumor cells [28, 39] with some similarity such as hypoxia response both enhanced aggressive phenotype [23, 28] and others had found some differences in mutant gene [40] which could lead CTCs to acquire more aggressive behaviors. These researches showed that CTCs not only acted as an intermediate, they also as the potential precursor cells of metastasis [41] during the movement of tumor cells from the primary site to a distant location and the establishment of a new cancer growth. Different environmental stresses lead to different fates of CTCs. Some special CTCs could survive by some phenotypic and functional alteration to resistant environmental stress [42]. More aggressive CTCs could become potentially tumor-initiating cells, but they were unique and heterogeneous cell populations by their relation to a series of biological processes, such as EMT or mesenchymal-epithelial transition (MET), differed from the CSC-like cells in primary site as many researches previous described [35, 43–45]. These potentially tumor-initiating cells may not only infiltrate into distant sites, and also recruit some immunosuppressive cells, particularly tumor-associated macrophages (TAMs) to create a defensive shield and build the secondary niches [10, 12, 19]. The different cellular and intracellular interactions could cause totally different antitumor immune responses and metastatic prognoses [42, 45]. It could partially explain the source of heterogeneity of tumor metastasis and development in clinic. Recently a study investigated the different regrowth of the same CSCs population in primary and metastatic sites from a mouse model of colorectal cancer [46]. The authors found that the specific stem-cell subpopulation were eliminated by Lgr5^+^ target therapy in both primary and metastatic sites, but when the drugs treatment ceased, the two sites had different outcomes. In primary site, the tumor increased in the size and the specific stem-cell population reappeared, but in the metastatic site, there was no relapsed [46]. It could be explained that these CTCs, which had migrated through the bloodstream, could more contribute to act as the role of tumor-initiating cells and to drive metastasis formation, rather than act as the role of CSCs, which must have more self-renewal ability to maintain metastasis growth [46]. Conversely in primary site, the other cells may have the reversible ability and fulfilled stem-cell functions to fuel tumor regrowth [46, 47]. Thus, the target therapy might be more effective on metastatic site than primary site. Besides the difference between CTCs and primary tumor cells, the heterogeneity also exists in different CTC subpopulations. Malara N et al. even showed different biological behaviors in two expanded CTCs (eCTCs) subpopulations derived from patients with colon cancer. The eCTCs subpopulation expressed CXCR4^+^CK20^+^ were not tumorigenic but able to disseminate, and the other subpopulation expressed CD45^−^CD133^+^ were more tumorigenic. Patients with different prevalence of CTCs had different clinical outcomes [48]. Thus, on these basis of the CTCs heterogeneous composition, many researchers now do believe that traditional clinical treatment strategies might not be useful to patients with metastasis, because these treatment strategies often based on the pathological and molecular characterization of the primary tumor [49]. As current functional researches showed, CTCs should provide more useful resources for the mechanisms of metastasis formation [41]. The detailing of sufficient eCTCs in distinct subsets, by qualitative and quantitative measurement, might be useful to better define a personalized metastatic risk score [48] and lead to a better way in identification and isolation of metastasis-initiator cells for further clinical individual treatment decision regarding drug resistance [27] or prognosis [17].

## The expanded methods of CTCs for clinical individual application

CSCs are known to be highly chemo-resistant [50] and more tumorigenic capacity under special microenvironment such as hypoxia-inducible condition [51]. They are always the key subsets that cause the treatment failed in whole tumor disease. Many researchers attempted to use the viable eCTCs to extend the knowledge of CSCs and figure out the process of metastasis formation. The methods that could get the more qualified eCTCs for reliable study are very crucial. CTC- derived xenograft (CDX) models is one of the expanded methods in vivo. Ameri, K et al. [28] using CDX to build a murine- derived CTCs model, showed that CTCs had an enhanced aggressive phenotype under chronic hypoxia. Their results revealed the micro-environmental stress could select for cells with phenotypes alterations and contributes to increased metastases. Successful CDX models could not only better mimic biological environment, it also recapitulate each individual patient’s cancer pathology and yield results more predictive of subsequent activity in patients [52]. However, using human cell line to generate murine-derived CTCs had its inevitable defects, because these CTCs from immunodeficient mice were not perfectly adequate for human [52]. For example, taken CTCs from cardiac puncture rather than from venous sampling, the most important differences are: *i* 2-7 ml blood is the minimal volume to human but is lethal to mouse, thus the enriched CTCs numbers are not on the same scale, CTCs in equal volume from mouse must be significantly higher than human. *ii* The sites that CTCs were directly punctured from heart means cardiogenic derived circulation in mouse, differed from peripheral venous and arterial circulation [53] in patients. And after that, although various studies reported that xenografts of CTCs were successful in many solid tumors, it should be also noted that many CDXs could be only obtained from advanced stage patients with high CTC counts, and even these xenotransplantation in vivo must take a long time [52].

The extended methods of CTCs in vitro were also reported. Many researchers suggested that the short term-eCTCs could distinguish from healthy or inflammation-derived cells that were isolated and unable to survive and expand [27, 48]. However, the maintenance of CTC culture in vitro from human blood samples is a complicated task, because many CTCs have limited proliferation ability and senesced after a few cell divisions in many cultural conditions such as adherent monolayer culture [30]. Lack of efficient conditions for eCTCs in vitro had become a bottleneck in clinic applications. Nevertheless, one study reported a microfluidic technology, human-CTC culture after enrichment by CTCiChip [54] showed the practicability of ex-vivo short term eCTCs in clinical trials. CTCs could be isolated and expanded from blood samples of early stage lung cancer patients, including patients with stage I [54]. In order to facilitate CTC expansion, the authors used a 3D co-culture condition, they introduced tumor associated fibroblasts to construct a tumor microenvironment [54]. Therefore, their expanded approach had high success rates to further characterize the biology of CTCs. And the long-term CTC cultures in vitro were reported by Min Y et al. They established oligoclonal CTC cultures sustained for > 6 months. CTCs were isolated from six of 36 patients with metastatic luminal subtype breast cancers [30]. In their serum free culture condition, the isolated CTCs could be maintained as suspended status and could form multi-cellular clusters, which were also named spheroids [55]. The eCTCs as non-adherent spheres may properly reflect intrinsic properties of CSCs that remain viable in the bloodstream after loss of attachment to basement membrane [30]. Spheroid culture of CTCs as a representative in vitro could reflect CTC cluster formation and growth in vivo [55]. Similar report was published by Cayrefourcq L et al. - the first CTC-derived permanent cell line isolated from the blood of a colon cancer patient, these CTCs had been cultured for more than 1 year [31]. It is a wealth of current functional researches on the biology of CTCs and raise the new perspective for drug testing in vitro and in vivo. But these long-term culture must also require high CTC counts from the advanced stage patients and were low success rate. Notably, there were another phenomenon might explain the low success rate. In Fan X et al.’s paper, the authors studied on 2 common prostate cancer cell lines named LNCaP and PC3 as research tools. Their results showed that PC3 could be formed spheres in suspension culture but LNCaP were failed [56] in the same condition. This suggested that different tumor cell lines, due to their different growth biology, could not either survive or expand in same culture mediums and environments in vitro. Thus, to better understanding CTCs biology from different origins, researchers must consider the merits and drawbacks in different culture conditions and approaches for clinical individual therapy (Table 1).

**Table 1:** Merits and drawbacks in three different methods for CTCs expansion

Method	CDX	Short term	Long term
CTC number	High	Low	High
Patient origin	Advanced stage only	Early and advanced stage	Advanced stage only
Condition	Experimental animal	10% FCS medium	Defined serum-free medium
Sample origin	Organ-vasculature circulation	Peripheral venous or arterial circulation	Peripheral venous or arterial circulation
Character	Tumorigenic capacity evaluation; complex procedure and individual difference	Differentiation and limited proliferation ability with significant phenotypic alterations	Phenotype stable; maintaining the tumorigenicity in non-adherent status
Research purpose	Simulate microenvironment in vivo	Expand enough CTCs for downstream analyses	Enrich and expand CTCs to establish patient-derived cell lines for long-term research
Cost	High	Cheap	Moderate
Culture cycle	Several months	1-2 weeks	Several months −1 year
Successful rate	Low	Moderate	Low

## Optimize the current approaches of CTCs culture

Before strategies of CTC culture apply for clinical management, some problems should be concerned to address properly. The further characterization of the expanded CTC-derived cell lines must be required to define clearly, such as CTCs proliferated as tumor spheres always cultured in serum-free medium which were far from the conditions in vivo. How they differed from cells cultured from primary tumor biopsies or directly implanted into mouse models are concerned issues [30]. The other key technical problems are how to maintain CTCs phenotype and composition of population stable in culture. Some reports even hint that normal human mesenchymal stem cells (hMSC) are prone to genomic change and subsequent malignant transformation in long term culture [57]. Thus, CTC culture may be also meet the same situation that caused the genomic instability under various environmental stresses, especially long-term culture. 3D biomaterial for co-culture is an ideal way to solve this problem, which could maximize to mimic tumor physical and biochemical microenvironment by adding the different culture ingredients, i.e. growth factors, hormones, serums, matrix components, and growth factors. It also could facilitate CTC expansion [54]. Thus, 3D biomaterial could be considered to integrate the different culture methods for more realistic drug responses [58]. Furthermore, define and modify culture media supplements properly for different tumor cell lines are much important for CTC culture (Table 2).

**Table 2:** Various formulas of culture for different sample-derived CSCs and CTCs

Purpose	Cell origin	Culture material	Cell seeded concentration	Initial treatment	Medium	Added ingredients	Environment	Culture cycle	ref
CSC/SC Sphere formation	Bladder cancer cells	Ultra-low attachment surface (Corning)	6 × 10^3^ cells/well	6-well plates	Serum-free DMEM/F12 (Gibco)	20 ng/mL EGF(Invitrogen), 20 ng/mL bFGF (Invitrogen), 1% N_2_ (Invitrogen), 2% B27 (Invitrogen) and 1% penicillin-streptomycin (Hyclone)		2 w	[79]
	Pancreatic Cancer KPCL Cell Line	Ultra-low attachment plates (Corning)	Tumor tissue minced	Promote organoid formation in serum-free for 3 days	Serum-free DMEM/F12	0.5% methylcellulose, 1% N2 (Invitrogen), 2% B27 (Invitrogen), 20 ng/ml recombinant human EGF (Miltenyi Biotec) and 20 ng/ml recombinant human FGF-2 (Miltenyi Biotec), 5 μg/ml heparin (Sigma) and 1% penicillin/streptomycin (Invitrogen)		3 d	[80]
	Kidney cancer cell lines ACHN /CAKI-1 RCC	Ultra-low attachment plates (Corning)	500 cells/well	96-well plate; 100 μl SFDM/well; add 25 μl SFDM /well /day	Serum-free defined media (SFDM) low-glucose (1 g/l) DMEM	L-Glutamine, sodium pyruvate, Penicillin/Streptomycin (Wisent Inc), 20 ng/ml basic FGF, 20 ng/ml EGF, and B27 (Invitrogen, Grand Island, USA)		3 w	[81]
	Brain metastases tumor	Ultra-low attachment surface (Corning)	200 to 2 cells /well (limiting dilution)	100 μL of cNSC media in a 96-well plate	Complete NSC (cNSC) media	Complete NSC media is comprised of NSC basal media (1% N2 [Gibco], 0.2% 60 μg/mL N-acetylcystine, 2% neural survival factor-1 [Lonza], 1% HEPES, and 6 mg/mL glucose in 1:1 DMEM/F12 [Gibco]), supplemented with 1× antibiotic–antimycotic (Wisent), 20 ng/mL human epidermal growth factor (Sigma),20 ng/mL basic fibroblast growth factor (Invitrogen), and 10 ng/mL leukemia inhibitory factor (Chemicon)	37 °C, 5% CO_2_	7 d	[82]
	Mammary gland stem cells	Low-attachment culture plate (Corning)	Serial dilution; 5-2000/well	96-well plate;	MM+ medium	DMEM/F12 supplemented with 2% calf serum, 10 mmol/L HEPES, 20 ng/mL epidermal growth factor (EGF), 10 μg/mL insulin, 5% bovine serum albumin, 1:50 B27 (Invitrogen), 20 ng/mL, basic fibroblast growth factor (bFGF), and 10 μg/mL heparin and 100 μg/mL penicillin/streptomycin		7 d	[67]
	Breast organoids	50-mm low attachment plat (Corning)	2.5 × 10^5^ cells/well	Dissociated into single cells after 6–8 h into 6-well plates	Serum free DMEM/F12 media	10 ng/ml hEGF, 1 mg/ml hydrocortisone, 10 mg/ml insulin, 20 ng/ml bFGF, 4 ng/ml heparin (Sigma Aldrich), B_27_ (Invitrogen) supplemented with antibiotics		7 d	[83, 84]
	HCC1806/MCF10A	Ultra-low attachment plates(Corning)	5 × 10^3^ cells/well		Mammary epithelial growth medium (MEBM)	Serum-free mammary epithelial growth medium (MEBM) (Lonza), supplemented with B_27_ (Invitrogen), 20 ng/mL EGF and 20 ng/mL bFGF (BD Biosciences), and 4 μg/mL heparin (Sigma).		10–14 d	[85]
	Brain tumor cell lines			Cells grown as monolayers were transfered into serum-free medium	DMEM high glucose (Sigma)	Serum free stem cell medium: DMEM/F12 (70/30%), 2% B_27_ (Invitrogen), 5 ng/mL heparin (Sigma), supplemented with 20 ng/mL human recombinant epidermal growth factor (hrEGF; Invitrogen), and 20 ng/mL human basic recombinant fibroblast growth factor (bFGF; BD Bioscience)	37 °C, 5% CO_2_	4–5 w	[86]
	Gastric cancer cell (patient- derived)		A single cell in 96-well plate	Samples were subjected to mechanical /enzymatic dissociation	Neurobasal-A medium (Gibco, Camarillo, CA)	Neurobasal-A medium (Gibco, Camarillo, CA) supplemented with 2 mM L-glutamine, 120 lg/ml of penicillin, 100 lg/ml of streptomycin, B_27_, 50 ng/ml of EGF, and 50 ng/ml of FGF-2. For differentiation, 5% FCS was added to the media instead of growth factors.		10 days	[68]
	PC3 human prostate cancer cells	100 cm^2^ culture dishes	1000 cells/ml		DMEM/F12	Serum-free DMEM/F12 medium containing 20 ng/ml epidermal growth factor (EGF; R and D Systems, Minneapolis, MN), 5μg/ml insulin, 0.4% bovine serum albumin (Sigma, St. Louis, MO), and 2% B_27_ (Invitrogen, Carlsbad, CA)	37 °C; humidified atmosphere; 5% CO_2_		[56]
CSC 3D culture	GBM6 cell line	3D CHA scaffold culture	50,000 cells /scaffold; 12-well plates		DMEM	DMEM supplemented with 2.5% FBS and 1% penicillin/streptomycin	37 °C humidified atmosphere 5% CO_2_	14 d	[87]
CTC culture	Patients with metastatic CRC (stage IV)	Ultralow attachment plates (Corning)	N/A	In 24-well plates	M12 medium (1 mL/well)	M12 medium contains advanced DMEM/F12 (Gibco), 2 mmol/L of L-glutamine, 100 Unit/mL of penicillin and streptomycin, N2 supplement (Gibco), 20 ng/mL of epidermal growth factor (R&D) and 10 ng/mL of fibroblast growth factor-basic (R&D)		3 w (5 × 10^6^ cells)	[25]
	Patients with breast cancer		N/A	24- or 6-well plates for further growth, and subsequently into T75 tissue culture flasks	DMEM/F12	Stem cell culture medium (DMEM/F12 containing 5 mg/ml insulin, 0.5 mg/ml hydrocortisone, 2% B_27_, 20 ng/ml EGF, and 20 ng/ml FGF-2) for the first seven days, then switched to EpiCult-C medium from day 8 (STEMCELL Technologies Inc.) with 10% FBS and 1% penicillin/streptomycin and continued to grow in this medium until day 21. The medium used from day 22 on was DMEM/F12 plus 10% FBS and 1% penicillin/streptomycin solution (Regular M)	37 °C, 5% CO2,	0-7;8-21;>22d	[77]
	Patients with colon tumor		N/A		DMEM/F12	Sphere medium used was DMEM/F12- Heparin 0.5 U/ml, EGF 50 ng/ml, FGF 25 ng/ml, BSA 1%, penicillin–streptomycin solution 1%.		14 d (short term)	[48]
	Patients with colon tumor	Non adherent plates	N/A	Culture in 24 well and into T25 flasks for culture expansion	DMEM/F12; RPMI1640	DMEM/F12 containing insulin (20 μg/mL), 1% N_2_ complement, epithelial growth factor (EGF: 20 ng/mL), L-Glutamine (2 mM), fibroblast growth factor-2 (FGF2: 10 ng/mL) and 2% foetal calf serum for the first days (Medium 1). After a few weeks, the CTC culture was switched to another appropriate culture medium to improve the CTC cell growth (Medium 2: RPMI1640, Growth factors: EGF and FGF-2, Insuline-Transferine-Selenium supplement, L-Glutamine) under normoxic conditions (5% CO_2_)	Hypoxic conditions; 2% O2; 37 °C	A few months obtained billions of tumor cells	[31]
	Patients with lung cancer (early stage)	3D material: Collagen; matrigel; fibroblasts	N/A	3D co: a mix of collagen and matrigel and fibroblasts 3D mono: cultured only with gel; 2D co: cultured only with fibroblasts 2D mono: without any gel nor fibroblasts	RPMI1640	RPMI1640 (10% FBS and 1% Penicillin/Streptomycin) maintained under different culture conditions and cultured up to 7 days on the chip: 3Dco; 3Dmono; 2Dco; 2Dmono		14 d	[54]
	Patients with head and neck tumor	Non adherent spheroid microplates (Thermo Scientific, USA)	N/A	Isolated CTCs were cultured in 96F well	DMEM/F12	Culture medium containing Advanced DMEM/F12 with the following additives: 50 ng/mL EGF (Sigma), 5% *v*/v R-spondin 1, 10% *v*/v Noggin, 10 ng/mL FGF10 (Peprotech), 1 ng/ml FGF2 (Peprotech), 10 nM Nicotinamide (Acros), 0.5 μM A83–01 (Tocris), 10 μM SB202190 (Sigma Aldrich), 10 μM Y-27632 (Selleck Chemical), 1X B_27_ Additive (Invitrogen), 1.25 mM N-Acetyl-L-cysteine (Sigma-Aldrich), 2 nM Glutamax(Invitrogen), 10 mM HEPES (Sigma Aldrich), 1:100 v/v Primocin (Invivogen)	2% O_2_; 5% CO_2;_ 37 °C	14 d	[32]
	Patients with pancreatic/urothelial/urinary bladder/ prostate Cancer		N/A	6-well cultivation plate	RPMI1640	Isolated CTCs by size-based separation methodMetaCell®, and grown in FBS-enriched RPMI1640 (10%) for a minimum of 3-6 days;	37 °C, 5% CO2	14 d	[17, 33, 88–92]

## Conclusions

Many studies have thought CTCs as a noninvasive method could provide a new perspective [59], but only enumeration is not sufficient [15, 60], it may only reflect relative tumor burden or leakiness of tumor-associated vasculature [40]. The quantification of CTCs with their viability are of high value for clinical evaluation, these CTCs with potential CSCs feature generally represent the tumor metastases and could be as procurators to facilitate real-time monitoring during systemic therapies by sequential peripheral blood sampling. But researchers must also keep an eye on those dormant CTCs in PB. A few of them may become the precursors of metastases in distant sites which offer appropriate conditions for them [61]. Thus, the optimal culture conditions for CTC expansion will need to be also considered for these special CTCs subsets. By utilizing different 3D biomaterials to improve culture microenvironment are the better options, it could screen out the more pertinent CTCs subsets and acquire more realistic information for strategy of personal therapy.

## Abbreviations

CDX: CTC-derived xenografts
CSCs: Cancer stem cells
CTCs: Circulating tumor cells
DTC: Disseminated tumor cells
ECM: Extracellular matrix
eCTCs: Expanded CTCs
EMT: Epithelial-Mesenchymal Transition
IBC: Inflammatory breast cancer
MET: Mesenchymal-Epithelial Transition
MSCs: Mesenchymal stem cells
PB: Peripheral blood
Ph: Philadelphia chromosome
SCs: Stem cells
SFM: Serum-free medium
TAMs: Tumor-associated macrophages
TICs: Tumor-initiation cells
